# Acute Lymphoblastic Leukaemia in the Youngest: Haematopoietic Stem Cell Transplantation and Beyond

**DOI:** 10.3389/fped.2022.807992

**Published:** 2022-02-24

**Authors:** Adriana Balduzzi, Jochen Buechner, Marianne Ifversen, Jean-Hugues Dalle, Anca M. Colita, Marc Bierings

**Affiliations:** ^1^Clinica Pediatrica Università degli Studi di Milano-Bicocca, Fondazione Monza e Brianza per il Bambino e la sua Mamma, Monza, Italy; ^2^Department of Pediatric Hematology and Oncology, Oslo University Hospital, Oslo, Norway; ^3^Copenhagen University Hospital Rigshospitalet, Copenhagen, Denmark; ^4^Hôpital Robert Debré, GH AP-HP. Nord Université de Paris, Paris, France; ^5^Department of Pediatric Hematology and BMT, Fundeni Clinical Institute, “Carol Davila” University of Medicine, Bucharest, Romania; ^6^Princess Maxima Centre, Utrecht, Netherlands

**Keywords:** haematopoietic stem cell transplantation (HSCT), acute lymphoblastic leukaemia (All), children, chimeric antigen receptor T-cells (CAR T cells), infants, total body irradiation (TBI), blinatumomab, FORUM trial

## Abstract

The ALL SCTped 2012 FORUM (For Omitting Radiation Under Majority age) trial compared outcomes for children ≥4 years of age transplanted for acute lymphoblastic leukaemia (ALL) who were randomised to myeloablation with a total body irradiation (TBI)-based or chemotherapy-based conditioning regimen. The TBI-based preparation was associated with a lower rate of relapse compared with chemoconditioning. Nevertheless, the age considered suitable for TBI was progressively raised over time to spare the most fragile youngest patients from irradiation-related complications. The best approach to use for children <4 years of age remains unclear. Children diagnosed with ALL in their first year of life, defined as infants, have a remarkably poorer prognosis compared with older children. This is largely explained by the biology of their ALL, with infants often carrying a *KMT2A* gene rearrangement, as well as by their fragility. In contrast, the clinical presentations and biological features of ALL in children >1 year but <4 years often resemble those presented by older children. In this review, we explore the state of the art regarding haematopoietic stem cell transplantation (HSCT) in children <4 years, the preparative regimens available, and new developments in the field that may influence treatment decisions.

## Introduction

Risk-adapted treatment stratification is the basis for modern paediatric acute lymphoblastic leukaemia (ALL) treatment. In general, children with ALL are considered eligible for haematopoietic stem cell transplantation (HSCT) when a dismal outcome is expected with standard chemotherapy (1). Early response to treatment is monitored by repeated measurements of minimal residual disease (MRD) based on either molecular sequences measured using polymerase chain reaction (PCR) or immunological cell surface markers measured using flow cytometry. Pre-defined MRD cut-offs are well-accepted for therapy stratification (1). See also the companion paper by Merli and colleagues in this supplement of Frontiers in Pediatrics.

In addition to suboptimal therapy response, other features of poor prognosis have been identified that stratify patients in most protocols to treatment intensification by HSCT (see companion paper by Truong and colleagues in this supplement). The most notable genetic lesions in leukaemic cells associated with a very poor prognosis are hypodiploidy, clonal abnormalities involving the *KMT2A* gene (previously known as *MLL*), TP53 alterations and the rare *t*(17;19) translocation, responsible for the *TCF3-HLF* fusion gene (1, 2).

In the case of relapsed disease, the timing and site of relapse, immunological lineage, as well as early treatment response after relapse (also defined by MRD analysis), are well-accepted factors used for defining an indication for HSCT (1).

An arbitrary threshold of 1–2 years of age has been historically used to determine the eligibility of paediatric patients for total body irradiation (TBI)-based myeloablative conditioning therapy prior to HSCT, as the younger the patient the more severe are the long-term side effects expected from radiation (3–5). Such an age threshold was raised to 4 years of age within the ALL SCTped 2012 FORUM (For Omitting Radiation Under Majority age) trial, according to which children ≥4 years with ALL eligible for HSCT were to be randomised between TBI-based and chemotherapy-based myeloablative conditioning, whereas children <4 years were allocated to the chemoconditioning arm in order to spare a larger proportion of the youngest children from the late effects of TBI (6).

The underlying biological features of ALL in children <4 years are very diverse, ranging from initial standard-risk features [i.e., B-cell precursor (BCP) ALL with *t*(12;21) translocation] to very high-risk features such as *KMT2A* gene rearranged ALL in infants, with HSCT being often indicated in first complete remission (CR1). Transplant indications in patients >1 year of age are, in general, not differentiated by age; however, treatment results vary by age, as described in more detail later (1).

Infant ALL (i.e., ALL diagnosed below the age of 1 year) is characterised by hyperleukocytosis, organomegaly, more frequent central nervous system (CNS) involvement, worse prognosis and substantially higher risk of early treatment-related mortality (TRM) compared with older children. Infant patients often require several therapy modifications due to toxicity compared with older patients (7).

Three main factors potentially influence HSCT indications in children ≤4 years old: disease biology, treatment-related toxicity during initial therapy, and the conditioning regimen. In addition, several novel therapeutic approaches that are available for the treatment of paediatric ALL may have a major impact on the decision making process, by decreasing early toxicity in the youngest patients, allowing HSCT to be performed with fewer and less-severe complications in this very fragile population with poor prognosis (7). Novel agents include blinatumomab (a bispecific CD3/CD19 antibody), inotuzumab ozogamicin (a toxin-conjugated anti-CD22 antibody), and chimeric antigen receptor (CAR) T-cell therapy, besides additional compounds, such as daratumumab and isatuximab (both anti-CD38 antibodies), nelarabine (a purine nucleoside analogue pro-drug) and venetoclax (a bcl-2 inhibitor) (2). Specific experiences with these approaches in this age group are mostly very limited.

The target of this paper is to review and summarise the state of the art and discuss unmet needs for patients below 4 years of age affected by ALL.

## Conventional Strategies Including Haematopoietic Stem Cell Transplantation

### Infants

#### Results of Frontline Trials

Children diagnosed with ALL in their first year of life (defined worldwide as infants) have a remarkably poorer prognosis compared with older children; this is largely explained by the biology of their ALL, as ~75–80% of them carry a *KMT2A* gene rearrangement. An event-free survival (EFS) of 50% or lower is reported in this age/biological group.

The cytogenetic hallmark of infant ALL is rearrangement of the *KMT2A* gene, previously called *MLL* (mixed lineage leukaemia), located at chromosome 11q23. *KMT2A* rearrangement originates from the fusion of *KMT2A* with a partner gene, resulting from translocations or other chromosomal rearrangements (2). The *KMT2A-AFF1* fusion (previously *MLL-AF4*) is the most common fusion of *KMT2A*, accounting for approximately 50% of cases. Almost 100 other fusion partner genes to *KMT2A* have been identified so far.

Children below 1 year old at diagnosis are treated, in general, according to specific protocols because of the particular biology and fragility of the infant patient. An HSCT indication in CR1 is still not uniformly settled in this age group, with most high-risk infants being often stratified to HSCT, according to age at diagnosis (usually <6 months), a *KMT2A* rearrangement, high leukocyte count (>300 10^9^/L) and/or poor prednisone response, in European, but not US protocols.

Data from previous trials including infants with ALL undergoing HSCT reveal a considerable risk of relapse (around 30%) and toxicity, with TRM around 20%. Highlights are reported in Table 1 (8–14). Sison and Brown presented a clear mini review of the available literature (16 articles) in 2013 (15). In general, many studies described infant cohorts treated several decades ago and often included limited patient numbers treated with non-homogeneous transplant procedures. More recent data are also given below.

**Table 1:** Results of trial protocols using HSCT in infants with ALL.

**Consortium**	**Treatment years**	**Pt age**	***N***	**Fraction w HSCT in CR1**	**EFS**	**OS**	**References**	**Comments**
Japan (JPLSG)	2011–15	<1	90	42%	71 (3-y, SE 4.9)	85 (5-y, SE 3.9)	(8)	43 of 49 eligible HR pt w HSCT in CR1, of these 67% alive. HSCT eligibility: KMT2A rearrangement, <6 months old, WBC >300 ×10^9^/L or PPR
Argentina	1990–18	<1	116	9%	32 (5-y, SE 4.6)	34 (5-y, SE 4.6)	(9)	Retrospective. Twenty-four percentage death in CR1, 42% relapsed. MRD and MLL risk factors for failure
Interfant 06	2006–16	<1	651	18%	48 (4-y, SE 2.0)	59 (4-y, SE 2.0)	(10)	54 of 143 HR-patients experienced an event before HSCT in CR1. 4-y DFS in all transplanted infants 44%, 14% died of TRM
COG	2001–06	<1	147	0%	42 (5-y, ±6%)	53 (5-y, ±6.5%)	(11)	Cohort 3 only. No HSCT in CR1 according to protocol
Japan (JPLSG)	2004–09	<1	62	85%	43 (4-y, 95% CI 31–55)	67 (4-y, 95% CI 54–77)	(12)	Only HR-pts, all w HSCT indication. Bu/Cy/VP16. 18/43 relapsed
Interfant 99	1999–05	<1	482	8%	47 (4-y, SE 2.6)	55 (4-y, SE 2.7)	(13)	HSCT in CR1 if PPR and available donor. DFS in HSCT group 50 vs. 37 in non HSCT pts (n.s)
Japan (JPLSG)	1995–02	<1	102	49%	51 (5-y, ±9.9%)	61 (5-y, ±9.8%)	(14)	20/74 in HSCT arm relapsed, one TRM before HSCT. 27/49 HSCT patients in CR1. Fifty percentage had TBI; 50% had Bu-based conditioning. No difference in outcome

*Bu, busulfan; CI, confidence interval; COG, Children's Oncology Group; CR1, first complete remission; Cy, cyclophosphamide; DFS, disease-free survival; EFS, event-free survival; HSCT, haematopoietic stem cell transplantation; HR, high risk; JPLSG, Japanese Pediatric Leukemia/Lymphoma Study Group; MLL, mixed-lineage leukaemia; MRD, minimal residual disease; OS, overall survival; PBC, Pediatric blood and Cancer; PPR, prednisolone poor response; SE, standard error; TBI, total body irradiation; TRM, transplant related mortality; VP16: etoposide; WBC, white blood cell; yr, year*.

A trial in 17 infants with ALL in CR1 conditioned with TBI (13.5 Gy in most cases, combined with cyclophosphamide) prior to HSCT in 1982–2003 was described by Sanders et al. Overall survival (OS) was 79%, with apparently mild-to-moderate long-term toxicity at a median post-transplant follow-up of 6 years (maximum 17 years), throughout which growth, endocrine and neuropsychiatric development disorders were most common (16).

No survival advantage of transplant was found by Dreyer et al. in a US cohort of infants with ALL treated in 1996–2000 who were allocated to HSCT or chemotherapy based on the availability of a suitable donor. The 5-year EFS rate was 49% for both the 53 infants who underwent HSCT and the 47 infants treated with chemotherapy only (17). Despite several study limitations, including small patient numbers and the analyses being run by treatment performed and not by intention to treat (17), the lack of benefit of HSCT in the cohort was the basis for chemotherapy-based treatment without HSCT being selected in a subsequent study cohort and for most infants with ALL in North America (7).

Attempts to improve outcomes by performing HSCT for all infants with *KMT2A*-rearranged ALL have been carried out in Japan through three consecutive clinical trials, but evidence has emerged that HSCT did not benefit every infant with ALL. The use of early HSCT in 62 infants with ALL in Japan in the late 1990s who were treated with short-course intensive chemotherapy and transplanted within 4 months of induction yielded a 4-year EFS of 43% and OS of 67%. The high relapse rate observed in the trial jeopardised the efficacy of the early HSCT approach, with the further limitation that pre-transplant MRD was not systematically studied in this cohort (12). Within the MLL-10 trial, enrolling 90 infants between 2011 and 2015 in Japan, 3-year EFS and 3-year OS rates for the 75 patients with *KMT2A*-rearranged ALL were 66.2% [standard error (SE), 5.6%], and 83.9% (SE, 4.3%), respectively, overall. The multivariable analysis showed that MRD at end of consolidation was the most powerful predictor of unfavourable EFS with a hazard ratio of 82.96 (8). High risk infants were eligible for transplant. Of the 56 high risk infants enrolled on study, 38 received HCST according to protocol and 5 received HSCT off protocol, with 29 of these 43 patients alive in first CR (8).

In 472 infants transplanted for malignant diseases in 2000–2014 and reported to the Centre for International Blood and Marrow Transplant Research (CIBMTR), 182 of whom had ALL, Parikh et al. observed no improvement in the outcome measures over time, as opposed to the general improvement of transplant results for most other ages and disease groups over time. Both the rate of relapse and the rate of toxicity remained high, with a high incidence of sinusoidal obstruction syndrome (SOS) (18). These data illustrate the challenges in infant ALL with high risks of both toxicity and relapse.

**Figure 1:**
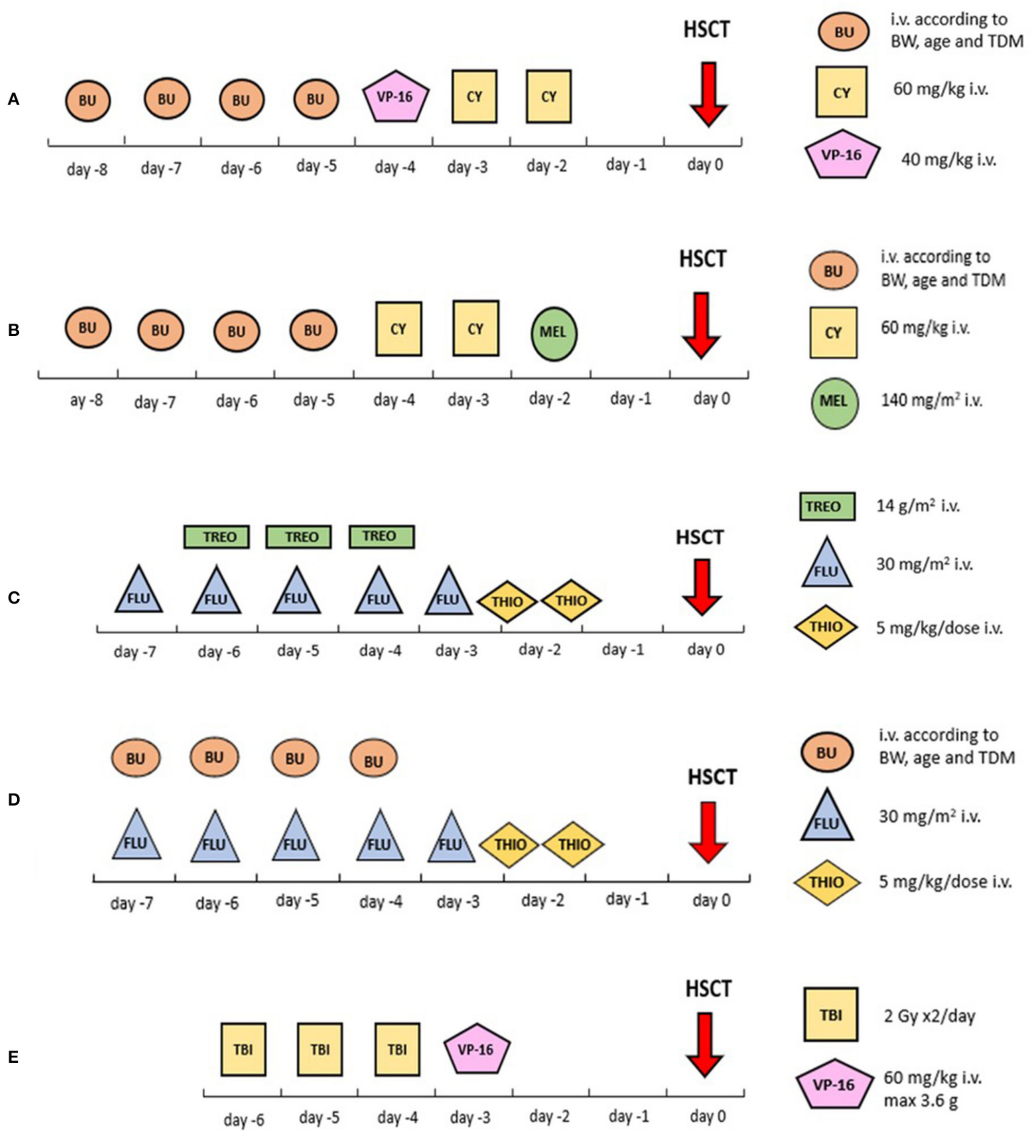
Visual summary of the most common conditioning regimens reported in this review. **(A)** Busulfan associated with cyclophosphamide and etoposide; **(B)** busulfan associated with cyclophosphamide and melphalan; **(C)** treosulfan associated with fludarabine and thiotepa; **(D)** busulfan associated with fludarabine and thiotepa; **(E)** TBI plus etoposide. BU, busulfan; CY, cyclophosphamide; FLU, fludarabine; HSCT, haematopoietic stem cell transplantation; Gy, Grey; HSCT, hematopoietic stem cell transplantation; i.v., intravenous; MEL, melphalan; TBI, total body irradiation; TDM, targeted drug monitoring; THIO, thiotepa; TREO, treosulfan; VP-16, etoposide.

Within the Interfant-99 trial of frontline chemotherapy in ALL, enrolling patients diagnosed with ALL <1 year of age in 1999–2006, the recommended conditioning regimen for patients eligible for HSCT consisted of busulfan, cyclophosphamide and etoposide, as described by Mann et al. (Figure 1A). The survival advantage of the 37 transplanted patients vs. the 240 treated with chemotherapy only was restricted to a subgroup carrying at least two additional poor-risk features: being <6 months of age and either having a poor steroid response on day 8 of treatment or a high leukocyte count at diagnosis (>300 ×10^9^/L). HSCT resulted in a 64% reduction in the risk of failure due to either relapse or death in CR vs. chemotherapy alone (hazard ratio 0.36, 95% confidence interval, 0.15–0.86) (19).

Results from the subsequent Interfant-06 trial, running from 2006 to 2016, remained somewhat disappointing. Patients who had a *KMT2A* rearrangement and were younger than 6 months with a white blood cell (WBC) count of >300 ×10^9^/L or a poor prednisone response were defined as high-risk and were eligible for HSCT. The eligibility for HSCT in the trial was extended in June 2009 to include also *KMT2A*-rearranged patients older than 6 months (medium risk) with persisting high MRD levels at time point 5 after MARMA chemotherapy. The recommended conditioning regimen consisted of busulfan, cyclophosphamide and melphalan (Figure 1B). Between 2006 and 2011, 13 of 50 (26%) patients who underwent transplantation died of HSCT-related complications. In 2012 the conditioning regimen was changed from busulfan, cyclophosphamide and melphalan into fludarabine and thiotepa associated with either treosulfan or busulfan (Figures 1C, D). Subsequently, only 3 of 61 (5%) patients died in CR after HSCT (10). However, the relapse rate remained high in these patients despite transplantation, being 34% in high-risk patients and 50% in medium-risk patients; 18 and 6% of the patients, respectively, died of non-leukaemic death. Conversely, none of the 7 medium-risk patients who were eligible for HSCT due to MRD but who did not undergo HSCT survived (10). The 6-year EFS rate of patients in the high-risk group was 20.9% (SE, 3.4), with many early events meaning that only 46% of these patients were transplanted. *KMT2A* rearrangement was the strongest prognostic factor for EFS, followed by age, WBC count, and prednisone response (10). In total, treatment translated into heavier toxicity in the infant population compared to older children. For comparison, TRM in the Interfant-06 trial for transplanted patients was 14.4%, whereas TRM was 4–9% in the FORUM study in children 4 years or older (6).

#### Indications for Transplantation

Due to their very poor prognosis, patients younger than 6 months at initial diagnosis who present with a WBC count above 300 ×10^9^/L and poor prednisone response are allocated to HSCT in CR1 in the Interfant-06 protocol, as are medium-risk patients with poor molecular response at timepoint 5 (10). The previously reported poor results were a combination of early toxicity, leading to HSCT contraindication, and very high relapse rate with or without further HSCT (10).

The upcoming Interfant protocol is in its planning phase. Blinatumomab will for the first time be introduced into an infant frontline protocol to reduce chemotherapy-related toxicity and with the aim to allow more eligible patients to proceed to transplant. Based on the uncertain benefits of HSCT in infants, it would be indicated for all high-risk patients and those medium-risk patients who are MRD positive (>0.01%) after a first blinatumomab cycle or who have increasing MRD after the MARMA chemotherapy element (personal communication).

### Children Younger Than 4 Years

#### Results of Transplantation

The clinical presentation and biological features at initial diagnosis of ALL are not generally different in children younger than 4 years of age compared to older children. Therefore, the same treatment protocols apply and results are rarely reported separately for this age group. Most relevant results in children younger than 4 years of age are highlighted in Tables 2, 3 (20–30).

**Table 2:** Outcome of HSCT after conditioning regimens based on TBI in children with ALL.

**Consortium**	**Treatment years**	***N***	**Patients 1–4 yr, n**	**Endpoint**	**Conditioning**	**EFS**	**OS**	**References**	**Comments**
AIEOP	1992–1997	40	13	3 yr	TBI-TT-Cy	CR1: 85%	65%	(20)	Better results in CR1. Study before 2000. Limited number of patients.
						CR2: 56%			
CIBMTR	1998–2007	765	NA	5 yr	Cy-TBI ≤ 1,200 cGy		44%	(21)	TBI ≥ 1,300 cGy associated with higher TRM.
					Cy-VP16-TBI ≤ 1,200 cGy		40%		
					Cy-TBI ≥ 1,300 cGy		48%		
					Cy-VP16-TBI ≥ 1,300 cGy		36%		
JSHCT (ALL working group)	2000–2012	767	NA	5 yr	Cy-TBI	62.2%		(22)	MEL-TBI: superior EFS for HSCT from MSD.
					MEL-TBI	71.4%			
					Cy-VP16-TBI	67.6%			
					Cy-AraC-TBI	52.6%			
					Others-TBI	59.1%			
I-BFM ALL-SCT-2003 trial	2003–2011	411	NA	4 yr	<2 yr: Bu-Cy-VP16 >2 yr: TBI-VP16	MSD: 79%	MSD: 80%	(23)	Lower TRM for MSD recipients.
						MUD: 71%	MUD: 78%		
I-BFM ALL-SCT-2007 trial	2007–2013	438	NA	4 yr	<2 yr: Bu-Cy-VP16 >2 yr: TBI-VP16	MSD: 65%	MSD: 72%	(24)	
						MD: 61%	MD: 68%		
I-BFM-ALL-SCT 2003 & 2007	2003–2013	1,150	69 (0–4 yr)	4 yr	<2 yr: Bu-Flu-Cy >2 yr: TBI-Flu-VP16	MSD/MD: 69%	MSD/MD: 60%	(25)	
						MMD: 45%	MMD: 42%		
Houston, USA	2008–2016	124	NA	3 yr	TBI-Cy-AraC <1 yr: Bu-based regimens	MSD: 63%		(26)	Single-centre experience. Similar outcome for MRD-negative patients regarding donor type.
						MUD: 58%			
						Haplo: 35%			

*AIEOP, Associazione Italiana di Ematologia e Oncologia Pediatrica; ALL, acute lymphoblastic leukaemia; AraC, cytarabine; BFM, Berlin-Frankfurt-Münster; Bu, busulfan; CIBMTR, Center for International Blood and Marrow Transplant Research; Cy, cyclophosphamide; CR1, first complete remission; CR2, second complete remission; EFS, event-free survival; Flu, fludarabine; Haplo, haploidentical donor; HSCT, haematopoietic stem cell transplantation; JPLSG, Japanese Pediatric Leukemia/Lymphoma Study Group; JSHCT, Japan Society for Hematopoietic Cell Transplantation; MD, matched donor; MEL, melphalan; MMD, mismatched donor; MSD, matched sibling donor; MUD, matched unrelated donor; MRD, minimal residual disease; NA, not applicable; TBI, total body irradiation; TRM, transplant-related mortality; TT, thiotepa; VP16, etoposide*.

**Table 3:** Outcome of transplantation after conditioning regimens based on chemotherapy only in children with ALL.

**Consortium**	**Treatment years**	***N***	**Patients 1–4 yr**	**Endpoint**	**Conditioning**	**OS**	**References**	**Comments**
Iran	1991–2011	183	NA	5 yr	Bu-Cy ± cranial irradiation	CNS positive: 51.9%	(27)	
						CNS negative: 47%		
Europe	2014–2015	65	NA	3 yr	Treo-Flu ± TT	73.8%	(28)	Included patients with ALL, AML, MDS or JMML. Higher OS for patients <2 yr.
Japan	2001–2003	10	1		AraC-Flu-MEL	80%	(29)	Case series report.
EBMT	2000–2012	3,054 (424 TBI free)	NA	5 yr	Fractionated TBI	CR1: 68.8% CR2: 58.8%	(30)	Comparative study of fractionated TBI- based and CC-based regimens. Bu-Cy most commonly applied in CC group. Significantly higher relapse rate with CC in CR2. Relapse rate and TRM were superior with TBI-based regimens vs. CC approaches.
					Bu-Cy* Bu-Cy-VP16 Bu-AraC ± MEL Bu-Cy-MEL Bu-Flu Bu-Cy-TT Bu-Flu-TT	CR1: 74.1% CR2: 35.9%		

*ALL, acute lymphoblastic leukaemia; AML, acute myeloid leukaemia; AraC, cytarabine; Bu, busulfan; CC, chemoconditioning; Cy, cyclophosphamide; CR1, first complete remission; CR2, second complete remission; EBMT, European Society for Blood and Marrow Transplantation; Flu, fludarabine; HSCT, haematopoietic stem cell transplantation; JMML, juvenile myelomonocytic leukaemia; MEL, melphalan; MDS, myelodysplastic syndrome; TRM, transplant-related mortality; Treo, treosulfan; TT, thio-tepa; VP16, etoposide*.

For 40 years, the combination of TBI, usually consisting of 12 Gy divided into 6 fractions, has been considered as the standard myeloablative conditioning regimen for children with ALL, most often in combination with cyclophosphamide (120 mg/kg divided over 2 days).

Since 1995, another TBI-based myeloablative conditioning regimen has been investigated for children 1 year of age or older, namely TBI in combination with etoposide (60 mg/kg as a single dose) (Figure 1E). An advantage of HSCT over chemotherapy could be demonstrated in very high-risk ALL patients after randomisation by the genetic chance of the availability of a compatible related donor (31).

Within the Berlin-Frankfurt-Münster (BFM) group (ALL SCT 2003 study) and, subsequently, the International-BFM Study Group (ALL SCT 2007 study), the TBI plus etoposide regimen was adopted for HSCT in patients 2 years or older (Figure 1E), whereas children younger than 2 years were conditioned with a TBI-free combination of busulfan, cyclophosphamide and etoposide (Figure 1A; body-weight-adjusted busulfan given orally or intravenously with dose monitoring and adjustment according to levels, every 6 h on days −7 through −4 for a total of 16 doses; cyclophosphamide 60 mg/kg/dose on days −3 and −2; and etoposide 40 mg/kg on day −1) (23–25).

Patients with a *KMT2A* rearrangement, regardless of age and based on their immature clonal phenotype, were eligible for an acute myeloid leukaemia (AML)-oriented conditioning regimen, consisting of busulfan, cyclophosphamide and melphalan (140 mg/m^2^ as a single dose on day −1) (Figure 1B) (24).

Overall, in the transplanted patients ≤2 years old, 4-year EFS was 67% (SE, 27%) for those grafted from a matched sibling donor (MSD) and 33% (SE, 16%) for those grafted from a matched donor (MD) (*p* = 0.2), whereas the 4-year non-relapse mortality was 0 and 33%, respectively (24).

The overlapping period between the transplant-specific BFM ALL-SCT-2003 and I-BFM ALL-SCT-2007 studies and the two infant chemotherapy trials, which were activated with different timings throughout centres, and the interaction between age and the presence of *KMT2A* rearrangements explained the multiple conditionings received by the youngest children, with busulfan, cyclophosphamide and etoposide being the treatment of choice according to Interfant 99 and the ALL-SCT trials and busulfan, cyclophosphamide and melphalan being the treatment of choice according to the Interfant 06 trial and overall for patients carrying a *KMT2A* rearrangement (19, 23, 24). Thus, the optimal conditioning therapy for children <4 years could not be defined due to the limited patient numbers and the lack of specific studies.

The phase III FORUM trial, comparing TBI plus etoposide vs. either a busulfan- or treosulfan-based myeloablative chemoconditioning in children (Figures 1C–E), raised the age cut-off for TBI eligibility up to 4 years. The FORUM study demonstrated the superiority of the TBI-based regimen compared with two chemoconditioning regimens (6).

However, patients <2 years old in BFM 2003 and I-BFM 2007 and patients <4 years old in the FORUM trial who were ineligible for randomisation were allocated to chemoconditioning upfront, since TBI in younger ages was felt to induce unacceptably severe multiple-organ long-term dysfunctions and neurocognitive abnormalities in survivors, being most pronounced in the youngest children (6, 16, 32, 33).

Results from the FORUM trial in children ≥4 years old demonstrated that omitting TBI from the conditioning regimen translated into an increased relapse risk (6). FORUM results on patients <4 years have not been analysed yet but may influence HSCT indications in young children. Whether TBI should remain excluded from the conditioning regimen of children 2–4 years of age may remain an object of discussion.

#### Indications for Transplantation

As described above, indications for HSCT in young patients, other than infants, are usually the same as those for children 4 years or older. For patients in CR1, these indications are mainly based on MRD response, monitored either by flow cytometry or reverse transcription PCR (RT-PCR). The algorithm for an HSCT indication may include biological and molecular features at initial diagnosis. HSCT indications are further discussed in the companion paper by Troung and colleagues in this supplement.

Some study consortia, such as FORUM, differentiate the indication for HSCT according to the donor type available [human leukocyte antigen (HLA)-identical sibling vs. other donors] and degree of HLA-matching (fully matched vs. partially matched donor), amongst patients with very high risk features, with patients carrying the best risk profile being eligible for HSCT from matched donors only and patients at highest risk profile being eligible for HSCT from any donor (<9/10 HLA compatible donor or 6/8 cord-blood and haploidentical donor) (23–25).

Currently, according to the IntReALL 2010 protocol, all patients in CR2 are eligible for HSCT from any available donor, except patients relapsing late in extramedullary sites.

The use of haplo-identical HSCT in this setting still remains controversial, as consolidated data about youngest patients are missing. Most paediatric reports, mainly retrospective and monocentric, were published without specific details about the youngest patients and it is thus difficult to draw conclusions. A Chinese group described better results in a HSCT cohort transplanted from haploidentical donors (*n* = 37) vs. tyrosine kinase inhibitor (*n* = 24) in high-risk paediatric patients with Philadelphia positive ALL. For the 14 patients <10 years, being younger than 10 year-old was associated with increased OS and EFS and lower TRM (34, 35). The same group published the results of haploidentical HSCT in 38 paediatric patients presenting with KMT2A rearranged ALL in either CR1 or CR2 but excluding infant patients. Overall results were comparable to those reported in the FORUM study within the MSD and MUD setting for the patients undergoing HSCT and significantly better than those obtained in non-transplanted patients. Authors used age 7 as cut-off prognostic factor without any impact on overall outcome (36). Readers may refer to the seminal paper about haplo-identical HSCT from Arrifin et al. in the same issue.

### Conditioning Regimens

#### TBI-Based Conditioning

Multiple TBI-based conditioning regimens have been adopted over time and throughout cooperative groups in paediatric ALL. Highlights are reported in Table 2.

The conditioning regimen TBI [9.9–12 Gy total dose, divided over 3 consecutive days (days −7 to −4)], thiotepa (10 mg/kg in 2 doses on day −4) and cyclophosphamide (60 mg/kg/day on days −3 and −2) was prospectively evaluated in 40 paediatric ALL patients by the Associazione Italiana Ematologia Oncologia Pediatrica (AIEOP) in the late 1990s and yielded a 3-year EFS of 85% for patients in CR1 and 56% for those in CR2. In the subgroup of patients aged 1–4 years, nine of 13 patients were alive at the end of the study period (20).

A study conducted by Tracey et al., including patients with ALL aged ≤18 years old, concluded that neither a TBI dose in excess of 13 Gy nor the addition of etoposide to cyclophosphamide could improve OS after HSCT but did increase TRM (18). TRM, as expected, was generally higher in patients >10 years old compared to in patients <10 years old (21).

The outcome of HSCT after multiple TBI-based conditioning regimens was retrospectively analysed in 767 ALL patients (in CR1 or CR2) by Kato and colleagues. In the HLA compatible setting, TBI both in combination with cyclophosphamide (120 mg/kg) and etoposide (30–60 mg/kg) or with melphalan (180–200 mg/m^2^) provided superior EFS rates compared with other regimens. The etoposide-containing regimen yielded a lower relapse rate and a non-significant increase in TRM while the melphalan-containing regimen yielded the lowest risk of relapse overall, despite an increased risk of TRM (22).

The ALL-SCT-BFM-2003 trial, as described above, included 411 paediatric ALL patients who underwent HSCT from either an MSD or MUD matched at 9 or 10 out of 10 HLA loci. Patients were stratified into 4 subgroups: 0–2, 2–12, 12–18, and >18 years old. The conditioning regimen used differed by age: patients ≥2 years received fractioned TBI (12 Gy in 6 fractions over 3 days) and etoposide (60 mg/kg) (Figure 1E); children <2 years old or children with contraindications to TBI (e.g., CNS irradiation before HSCT) were treated with intravenous busulfan with therapeutic drug monitoring plus cyclophosphamide (120 mg/kg total dose) plus etoposide (40 mg/kg total dose) (Figure 1A). OS, EFS and relapse incidence were similar for patients who had an MSD or MUD, but lower TRM was observed for MSD recipients (23).

The I-BFM ALL-SCT-2007 trial, which extended to 10 countries, confirmed the non-inferiority of HSCT from an MUD compared with HSCT from an HLA-identical sibling, with no significant difference in OS, EFS, probability of relapse or TRM observed (24).

The use of mismatched grafts (compatibility <9 out of 10 HLA loci matched, including haploidentical grafts) yielded an inferior outcome (4-year OS, 56%), as assessed within the ALL SCT 2003 and 2007 merged studies of the I-BFM Study Group, compared with 69% in the ALL SCT 2003 and 70% in the ALL SCT 2007 (25).

A different approach to classical TBI-based conditioning was assessed by Yanir et al. in a study that included 124 paediatric ALL patients undergoing HSCT, 71 of whom were in the younger age subgroup of 1–10 years. The addition of arabinoside cytosine to a regimen of TBI (1,200 cGy for MSD and 1,400 cGy for unrelated donors) and cyclophosphamide allowed the reduction of the cyclophosphamide dose from 120 to 90 mg/m^2^, with an aim to reduce long-term toxicity. Serotherapy with anti-CD52 (alemtuzumab) was added for unrelated and haploidentical HSCT. Patients with contraindications to TBI received busulfan-based regimens. HSCT from an MSD or MUD yielded similar EFS (63 and 58%, respectively) and relapse incidence (20 and 24%, respectively). However, patients transplanted from a haploidentical donor had worse outcome, with an EFS of 35% and a probability of relapse of 47% (26).

As the main reasons to refrain from the use of TBI in young children are either the presence of comorbidities or toxicities from pre-HSCT therapies as well as the expected long-term toxicity associated with TBI, it remains difficult to assess from these studies whether the use of TBI in children over 2 years of age was warranted.

#### TBI-Free Conditioning Regimens

Similar to the case for TBI-based conditioning (see above), most reports regarding TBI-free conditioning regimens discussed here were not restricted to or separately analysed for patients <4 years of age but rather involved or reported a more extensive age group. Highlights are reported in Table 3.

A 5-year OS of 47% was reported in a study in Iran after a TBI-free conditioning regimen consisting of busulfan (1 mg/kg once daily on days −7 to −4; weight adjusted after 2009) plus cyclophosphamide (60 mg/kg/day on days −3 and −2) in 184 patients aged 18 years or younger undergoing peripheral blood HSCT from HLA-identical siblings between 1991 and 2011. Cranial irradiation (1,200–1,800 cGy) was applied before admission to the transplant unit for patients with intermediate-to-very-high risk T-cell ALL and very-high-risk B-cell ALL (27).

The inclusion of treosulfan into conditioning regimens for paediatric ALL is relatively recent. An early experience in 40 patients younger than 18 years who were affected with acute leukaemia and myelodysplastic syndrome (MDS), including 23 paediatric patients with ALL, was reported by Kalwak et al. The body surface area (BSA)-adapted conditioning was fludarabine 30 mg/m^2^/day on days −7 through −3, intravenous treosulfan on days −6 through −4 (10 g/m^2^/day for BSA <0.5 m^2^, 12 g/m^2^/day for BSA 0.5–1 m^2^ and 14 g/m^2^/day for BSA >1 m^2^) and thiotepa 10 mg/kg used at the investigator's discretion on day −2. In the full cohort, 3-year OS was 73.8% and the probability of relapse was 26.1%; both of these outcomes are comparable to data using a classic myeloablative regimen. Exploratory analyses of all included patients indicated that the OS was higher in the eight patients aged 28 days to 23 months (100%, 90% CI: 100–100%), compared with the 32 patients aged 12–17 years (74.9%, 90% CI: 59.5–85.1%) (28).

A busulfan- and TBI-free conditioning regimen in patients with high-risk acute leukaemia undergoing HSCT from unrelated donors was reported by Kato et al. The conditioning consisted of granulocyte-colony stimulating factor (G-CSF; 5 μg/kg) 12 h before cyclophosphamide (1–3 g/m^2^/day) on days −10 to −6, fludarabine (30 mg/m^2^/day) on days −9 to −6, and melphalan 60 mg/m^2^/day on days −5 to −3. In the full cohort, two patients relapsed and died, whereas the remaining eight survived (29).

Willasch et al. analysed outcomes of 3,054 transplants performed in children aged 2–18 with ALL between 2000 and 2012 reported to the European Society for Blood and Marrow Transplantation (EBMT) registry. Most patients were conditioned with a TBI-based regimen, combined most often with cyclophosphamide or etoposide. Chemotherapy-only regimens were mainly busulfan based, most often used in association with cyclophosphamide. TBI-based regimens led to superior survival for patients in CR2, compared with that obtained in patients in CR1. Both relapse rates and TRM were lower with TBI-based regimens vs. chemoconditioning approaches (30).

The allocation to each chemoconditioning arm within the FORUM trial was based on a decision taken upfront on a country level between busulfan vs. treosulfan use in association with fludarabine and thiotepa (Figures 1B, C). Preliminary analyses presented at the EBMT Meeting 2021 did not identify the superiority of one chemoconditioning regimen over the other, even when separately analysed by B or T immunophenotype (37).

A novel TBI-free conditioning regimen consisting of clofarabine, fludarabine and busulfan has been recently reported in 60 children affected with ALL in The Netherlands. The reported 2-year EFS of 72% and a 2-year TRM of 5% in ALL allow one to define such a strategy as effective and having low toxicity. Despite having only 9 ALL patients younger than 4 years—which is too limited to allow conclusions for this age group—this conditioning regimen deserves to be explored further, especially for infants, for whom the TRM rate with other conditioning regimens is still unacceptably high (38).

In general, these studies do not allow the identification of an optimal chemotherapy-based conditioning regimen in children 4 years or younger. This emphasises the need to analyse the non-randomised cohort of the FORUM trial, which is currently underway.

## Innovative Approaches

In all age groups, the primary cause of treatment failure after HSCT is relapse, thus a more efficient anti-leukaemic treatment prior to HSCT is warranted. Nevertheless, toxicity of increased treatment intensity is a limiting factor for its use, especially in the youngest patients. This makes the clinical management of younger patients particularly challenging. Further intensifying chemotherapy doesn't seem an option, thus new treatment modalities with lower toxicity aimed to bridge to HSCT are warranted. Many new drugs are being tested currently (2).

During the last decade, novel targeted immunotherapy approaches, e.g., blinatumomab, inotuzumab ozogamicin and anti-CD19 CAR T-cell therapy, have emerged. These novel strategies might offer the potential for improving cure rates in the youngest children by: (a) inducing deeper molecular remissions prior to HSCT; (b) substituting intensive chemotherapy and thereby reducing the burden of pre-transplant toxicity; and/or (c). potentially replacing the HSCT procedure with non-/less toxic targeted cellular strategies.

### Pre-transplant Immunotherapy

#### Blinatumomab

Blinatumomab—a CD3/CD19 bispecific T-cell engaging antibody—has been studied in several paediatric BCP ALL settings in different disease phases and age groups, including infants. However, the experience in the youngest age groups remains limited.

In 2011, Handgretinger et al. reported on the first clinical experience in three paediatric patients who received blinatumomab for BCP ALL relapses after HSCT (34). All three patients were >4 years of age and received blinatumomab after multiple relapses and allogeneic HSCT. This very first report on the use of blinatumomab in children demonstrated that blinatumomab could be safely administered to children. It also showed that engaging donor T cells post transplantation did not provoke graft-vs.-host disease (GvHD) and that blinatumomab was able to induce MRD responses even in patients with chemo-refractory disease after multiple relapses (39).

The first trial studying systematically the efficacy and safety of blinatumomab in children and adolescents was a phase I/II open-label, single-arm study performed at 26 study sites in Europe and the US (Clinicaltrials.gov: NCT01471782) (35). Eligible patients were <18 years of age and had relapsed or refractory (R/R) BCP ALL with >25% bone marrow blasts at enrolment. The BCP disease status was primary refractory, in first relapse after full salvage induction regimen, in second or later relapse, or in any relapse after allogeneic HSCT. Forty-nine patients were treated in phase I and 44 patients in phase II. Eight and two patients in these phases were <2 years of age, respectively. In phase I, the maximum tolerated dose of blinatumomab was determined to be 15 μg/m^2^/day for all age groups. The recommended phase II dose for all ages was determined as 5 or 15 μg/m^2^/day (1 week of 5 μg/m^2^/day followed by 3 weeks of 15 μg/m^2^/day during the first cycle and for all subsequent cycles). Among the 10 patients who were <2 years of age, 6 (60%) achieved CR (including five of the eight patients with *KMT2A* translocations), with 4 (40%) being able to proceed to HSCT while in CR. Overall, 39% of patients achieved CR within the first 2 cycles of blinatumomab, with most responders achieving complete MRD negativity. The study showed that blinatumomab had anti-leukaemic activity across all age groups, including in patients <2 years and in those with unfavourable cytogenetics (40).

In the blinatumomab expanded-access program (the RIALTO trial; Clinicaltrials.gov: NCT02187354), patients with a second or later relapse, any relapse after allogeneic HSCT, or who were refractory to other treatments received blinatumomab for 1–2 induction cycles with the option to receive up to three additional blinatumomab consolidation courses (36). In total, 110 patients were enrolled, of which 13 and 31 patients were in the age groups 0–1 and 2–6 years, respectively. At screening, 11% of all patients had <5% bone marrow blasts, while the remainder had ≥5%. Sixty-nine of the 110 study patients (63%) had CR as best response in the first 2 cycles; of these, 45 (65%) proceeded to HSCT. MRD response was dependent on the pre-infusion blast count, being 47 and 92% for patients with ≥5 or <5% blasts, respectively. No age-specific subgroup analyses were detailed for the age groups 0–1 or 2–6 years (41).

In a single-centre experience, outcomes for 38 patients treated with blinatumomab over a 10-year period were reported (42). All patients had R/R (first to fourth relapse) disease. Median age upon blinatumomab initiation was 9.8 years, ranging from 1 to 21 years; eight patients were in CR with MRD positivity and 30 patients had blast counts of >5%. Thirteen patients (34%) responded to therapy; patients aged 2–10 years responded more frequently (7 of 10) than older children or children 1–2 years of age.

A retrospective analysis from the United Kingdom and the Republic of Ireland focused specifically on the blinatumomab experience in patients initially diagnosed with BCP ALL at <1 year of age (43). The analysis included 11 patients with *KMT2A*-rearranged BCP ALL aged a median of 0.5 years (range 0.2–2.9 years) who were in first remission or first relapse and who received blinatumomab with the aim to reduce pre-transplant MRD. Nine of the 11 patients achieved molecular remission and 2 had at least a 1-log reduction in MRD as best response. All patients proceeded to HSCT after 1–2 cycles of blinatumomab, without further intervention. Time from start of blinatumomab to HSCT was 51 days (range 34–119). The treatment was well-tolerated, with three patients experiencing cytokine release syndrome (CRS) of grade 1–2 and one experiencing immune effector cell-associated neurotoxicity syndrome (ICANS) (confusion and somnolence). Three-year OS and EFS after HSCT were 47 and 81%, respectively. Of the four patients who relapsed after HSCT, one experienced a lineage switch to AML. The report concluded that blinatumomab can be safely administered in this young group of patients with R/R BCP ALL and is able to induce molecular remission in the majority of patients, allowing consolidation with HSCT (43).

Sutton et al. reported on the real-world experience of blinatumomab in Australia and included 24 children (mean age 7 years, range 0.5–16.5 years) (44). Ten patients were <4 years of age at blinatumomab infusion, 9 had *KMT2A* rearrangements, and 7 were <2 years of age. Patients received 1–2 cycles of blinatumomab with the intention to achieve a deep molecular remission as a bridge to a first, second or third HSCT. Of the 10 patients <4 years at infusion, 4 (40%) responded to blinatumomab with either a complete or partial MRD response. The authors discussed that the lower response rate compared to that reported by Clesham et al. (43) could be explained by a higher proportion of patients with >5% blasts and more patients having had post-HSCT relapse or having received extensive salvage regimens prior to blinatumomab, all impacting on CD3^+^ T-cell number and function. Genetic factors might also have influenced blinatumomab effectiveness, especially in infants and young children with a *KMT2A* rearrangement.

In a report from five North American paediatric centres, 15 patients in remission (10 CR1, 5 CR2) but with persistent MRD prior to HSCT received blinatumomab with the aim to reduce MRD (45). Median age was 9 years (range 0.5–19 years); five patients were <4 years old at blinatumomab infusion. No patient experienced grade 3 or 4 CRS; one patient experienced grade 3 ICANS. Of the five patients <4 years of age, four in CR1 responded (three of them had a *KMT2A* rearrangement) and one in CR2 did not respond.

Finally, two randomised phase III studies evaluating blinatumomab in patients with a first BCP ALL relapse were recently published back-to-back in the Journal of the American Medical Association (46, 47). Enrolment into each study was prematurely terminated by recommendation of the respective independent data monitoring committee due to significant better outcomes in the blinatumomab arm vs. the control arm. In the study by Locatelli et al., 108 patients were randomised following initial induction therapy and two consolidation blocks to receive either a chemotherapy consolidation block according to the IntReALL high-risk (IntReALL HR) 2010 protocol or 1 cycle of blinatumomab (4 weeks of 15 μg/m^2^/day) (46). Thirty-nine patients (72%) were in the age group 1–9 years. The 24-month EFS rate was 66.2% in the blinatumomab group and 27.1% in the consolidation chemotherapy group. More patients in the blinatumomab group than in the consolidation chemotherapy group were able to proceed to HSCT. The cumulative incidence of relapse 24 months after transplantation was 24.9% in the blinatumomab group and 70.8% in the consolidation chemotherapy group.

In a parallel study performed at Children's Oncology Group sites and reported by Brown et al., patients between 1 and 30 years of age with first B-cell ALL relapse were randomised after 4 weeks of UKALLR3 induction therapy to either receive 2 courses of blinatumomab or chemotherapy consolidation (47). Randomisation was prematurely stopped due to the combination of higher disease-free survival and OS, lower rates of serious toxicity, and higher rates of MRD clearance with blinatumomab compared with chemotherapy. Seven patients in the blinatumomab arm and 10 patients in the chemotherapy arm were <1 year of age at the time of initial diagnoses (relapse timepoint); however, no detailed subgroup analyses were presented for these patients or patients <4 years of age.

Brethon et al. reported an interesting case report where blinatumomab and gemtuzumab ozogamicin were combined in a 4-month-old child with *KMT2A*-rearranged, mixed-phenotype leukaemia (48). Subsequently, the child was transplanted, relapsed and achieved remission again with CAR T-cell therapy.

In summary, current evidences point towards the efficacy and manageable toxicity of blinatumomab in patient groups <4 years of age, specifically in the context of MRD-positive disease prior to HSCT and as a substitution for single chemotherapy blocks in clinical situations in which toxic and intensive chemotherapy needs to be avoided (e.g., severe infection and/or surgical interventions) (49). Challenges for blinatumomab therapy are lineage switch as an escape mechanism, treatment beyond CR1, and specific genetic alterations such as *KMT2A* rearrangements.

Whether moving blinatumomab to upfront therapy, as planned in the upcoming Interfant trial, might improve outcomes was preliminarily investigated in a single-arm pilot trial in infants treated according to Interfant-06. The study was conducted to test feasibility, safety and efficacy of the addition of blinatumomab after induction in infants with KMT2A-r ALL and with <25% medullary blasts at the end of induction (EudraCT: 2016-004674-17) (50). MRD negative CR occurred in 54% of the cases after 2 and 4 weeks of blinatumomab, which tended to be higher compared to the end of consolidation in Interfant-06 (40%, *p* = 0.16). The 1-year EFS was 96.2% (SE 3.8) at a median follow-up of 11 month (range 1.5–33).

#### Inotuzumab Ozogamicin

Inotuzumab ozogamicin is a CD22-targeted antibody–drug conjugate which in phase I and II studies in adults has shown a beneficial efficacy-to-toxicity ratio. In an adult phase III trial of 326 patients with R/R ALL, the drug was highly efficient with an overall response rate (ORR) of 81% in the inotuzumab ozogamicin arm vs. 29% in the standard-of-care chemotherapy arm (51).

So far, few data have been published in children. In a retrospective report summarising the experience from the paediatric compassionate use program (52), 51 patients aged 2.2–21.3 years (median 11.5 years) were treated with inotuzumab ozogamicin for R/R BCP-ALL between 2013 and 2016, with only three of them being 2–4 years of age. CR was seen in 67% of the patients who were treated for overt relapse; 71% of responders achieved MRD negativity in the bone marrow—in most patients after the first cycle. Responses were independent of age and no separate data for the three patients younger than 4 years were reported. However, the single patient with a *KMT2A* rearrangement in the cohort responded well and achieved MRD-negative CR. Inotuzumab ozogamicin was generally well-tolerated, even by patients who were heavily pre-treated by multiple lines of therapy. Twenty-one patients underwent HSCT after inotuzumab ozogamicin with a median time from last dose of inotuzumab ozogamicin to stem cell infusion of 26 days. Eleven of 21 patients (52%) developed post-HSCT SOS, with 5 and 2 being severe and fatal, respectively. The 12-month EFS and OS rates for the entire cohort were 23 and 36%, respectively.

In the Innovative Therapies for Children with Cancer in Europe (ITCC) phase I dose-finding study of inotuzumab ozogamicin, 25 patients (including five patients <6 years old), were included (53). Although safety (dose-limiting toxicity) was the primary endpoint, the overall remission rate across dosing levels was 80%, with 84% of the responders being MRD negative, comparable to results from adult studies. The one patient who had *KMT2A-*rearranged ALL responded to inotuzumab ozogamicin. Hepatotoxicity was the primary dose-limiting toxicity, with two patients experiencing SOS; however, this occurred not during inotuzumab ozogamicin therapy but during subsequent multi-agent chemotherapy for non-response. None of the seven patients who underwent HSCT post inotuzumab ozogamicin developed SOS. The recommended phase II dose for children was determined to be the same as for adults. In the adult cohort (51) and the paediatric compassionate-use cohort (46), SOS was more frequently seen than in the phase I study, both under inotuzumab ozogamicin therapy (adults) and during later HSCT (adult and paediatric cohort). One could speculate that the burden of overall toxicity from previous lines of therapy might have been different in these cohorts and contributed to the differences in SOS occurrence. In the same cohort, subgroup analysis showed that those who could undergo HSCT had superior outcomes whether MRD positive or MRD negative at HSCT, indicating that inotuzumab ozogamicin potentially is a relevant option to bridge to HSCT (54).

Data from a series of 15 patients with R/R BCP-ALL aged <3 years treated with inotuzumab ozogamicin were recently published (53). Of these, 12 patients were <1 year of age at the initial diagnosis of ALL (i.e., patients with infant ALL) and 80% had a *KMT2A* rearrangement. In all but 1 patient, inotuzumab ozogamicin was used as third-line therapy. Overall, seven patients (46.6%) achieved CR and one additional patient who was MRD positive at start of inotuzumab ozogamicin therapy achieved MRD negativity. Overall, seven of these eight responders were MRD negative. Seven patients proceeded to HSCT, of whom three were alive at a median follow up of 342 days (range 19–361 days) for the whole study. Two of the seven patients receiving HSCT developed SOS of which one case was fatal. No patient developed SOS while receiving inotuzumab ozogamicin. EFS and OS at 6 months were 18 and 47%, respectively. The authors concluded that further investigation of the drug is warranted in this age group. Of note, in neither of the two patients younger than 1 year of age upon inotuzumab ozogamicin infusion nor in any of four additional patients <10 kg at infusion were any specific safety concerns raised.

The increased risk of SOS is particularly relevant in this fragile population who are already at risk due to their age.

In summary, inotuzumab ozogamicin is a promising drug, currently best studied in the setting of residual MRD or refractory disease. With current HSCT strategies, preventive supportive care and close monitoring according to paediatric guidelines, SOS should be manageable in children. A systematic and prospective phase II study in children is currently ongoing (ITCC-059, EudraCT: 2016-000227-71) investigating inotuzumab ozogamicin both as monotherapy and in combination with chemotherapy for patients with high-risk and very high-risk relapsed BCP-ALL ≥1 and <18 years of age at the time of enrolment. Another study by the COG (ClinicalTrials.gov: NCT02981628) is investigating inotuzumab ozogamicin in combination with a chemotherapy backbone in patients 1–21 years old with R/R BCP-ALL. The upcoming IntReALL trial might plan to include inotuzumab ozogamicin as induction therapy in high-risk relapsed patients.

### Chimeric Antigen Receptor T-Cell Therapy

Immunotherapy with autologous T cells that have been genetically modified to express an anti-CD19-specific CAR is a very promising new approach to treat acute leukaemia. See also the companion paper by Buechner and colleagues in this supplement. A CAR T-cell strategy has the potential to: (a) replace HSCT for a fraction of patients that is yet to be defined, or (b) to induce a deep remission prior to HSCT, also with shorter CAR T cell living variants, possibly limiting the use of high-dose chemo- or radiotherapy conditioning regimens and potentially reducing the risks of severe acute and chronic GvHD. There are, however, limitations to its efficacy, including the development of CD19^−^ relapses or the premature loss of CAR T cells associated with a CD19^+^ relapse.

T-cell apheresis and manufacturing of CAR T cells in infants are challenging but feasible, as described by several groups (55–57). In a recent meta-analysis on 953 patients treated with tisagenlecleucel (Kymriah®) (58) (a CD19-directed CAR T-cell therapy which is approved and commercially available for R/R BCP-ALL in patients aged 1–25 years), no differences in outcome were seen across the different age groups. However, it must be emphasised that the ELIANA registration trial for tisagenlecleucel (ClinialTrials.gov: NCT02435849) (59), excluded patients <3 years of age. The youngest age group has been included per an amendment of the expanded access programme B2001X which followed subsequently. After the approval of tisagenlecleucel by the US Food and Drug Administration in August 2017 and by the European Medicines Agency in August 2018, data from real-world experience have more recently emerged (55, 57), but the number of patients under 3 or 4 years of age is still very limited and this age group is most often not specifically addressed in the reports. In a conference abstract by Moskop et al. (56), real-world data on 14 infants (80% of them with a *KMT2A* rearrangement) treated with tisagenlecleucel were reported. Although apheresis and manufacturing of cells were feasible, the outcome (64% of the patients achieved an MRD-negative CR at day 28) was slightly lower than that reported for older children, both in the real-world (55, 57) and in the earlier registration study (59). However, compared to standard-of-care chemotherapy approaches, and considering the fact that these patients had R/R disease, the outcomes are still promising and need further prospective and comparative investigations.

One concern of using CD19-targeted therapies, including CD19-directed CAR T-cell therapy, is the risk of lineage switch as an escape mechanism, especially in cells with a *KMT2A* rearrangement. Gardner et al. described two relapses among seven patients with *KMT2A*-rearranged leukaemia treated with CAR T-cell therapy (60). Both patients presented with a myeloid phenotype with a loss of expression of B lymphoid lineage antigens. Jacoby et al. have unravelled and described the molecular events during lineage switch following CD19-directed CAR T-cell therapy in detail (61).

Ghorashian et al. reported at the European Haematology Association conference 2021 a study in which 27 children younger than 3 years (median age 17 months) were infused with tisagenlecleucel out of 30 eligible patients, 80% of whom carried a *KMT2A* rearrangement and 70% of whom had undergone prior HSCT. Leukaphereses and product manufacturing were feasible in 90% of cases. Ninety-two percent of patients achieved CR (confirmed by negative MRD), 1-year OS was 88%, and EFS was 58%. Of the responding patients, 37% were in continuous CR after further treatment post CAR T-cell infusion, whereas 22% experienced relapse, which was CD19^−^ in 33% of the cases. The probability of persistent B-cell aplasia at 1 year was 68% and the probability of EFS without further treatment was 49%. Risks of CRS, severe CRS, ICANS or persistent cytopenia were similar compared with the other age strata (62).

Hu et al. reported a cohort of paediatric and young adult patients presenting with relapsed/refractory Philadelphia chromosome negative B-cell ALL. Among 81 screened patents, 75 were enrolled for receiving CAR-T cells as bridging therapy to haplo-HSCT. Seventy-three received CAR-T, 57 were transplanted, 52 of whom from haploidentical donor, with a median time of 62 days elapsing from CAR-T cell therapy to haplo-HSCT. With this combined treatment, the 2-year EFS and OS were 76.0% (95% CI, 64.2–87.7) and 84.3% (95% CI 74.3–94.3), respectively, with a cumulative incidence of relapse of 19.7% (95% CI 15.3–24.0) (63).

CAR T-cell therapy seems to have a favourable toxicity profile compared to conventional therapy, yet currently it is not clear in infants or in older children whether or how it could replace allogeneic HSCT or chemotherapy elements. It may also have a role in achieving deeper remissions before a consolidating transplant.

### Emerging Options for Managing T-Cell ALL

T-cell ALL is rare in the youngest children. However, drugs like nelarabine and daratumumab have been used to reduce MRD levels prior to HSCT in these patients.

In a phase III study from the COG, 323 patients who received nelarabine added to standard therapy had superior disease-free survival compared with 336 patients randomised to standard therapy without nelarabine (88 vs. 82%, *p* = 0.029) without any difference in neurotoxicity between arms (64). Whether HSCT following nelarabine adds to the risk of neurotoxicity remains unclear and should be taken into account in the planning phase (65).

Daratumumab—an unconjugated monoclonal anti-CD38 antibody—is currently being investigated in a phase II study for the treatment of children >1 year old with BCP- or T-cell ALL (ClinicalTrials.gov: NCT03384654). Both T-cell ALL and B-lineage ALL cells can overexpress CD38 and are potential targets for treatment with daratumumab, which has been shown to have efficacy in adult cancers, especially in CD38^+^ multiple myeloma. Since CD38 is expressed on haematopoietic stem cells, awareness of the long half-life of daratumumab has led researchers to question the applicability of the drug for bridging to HSCT but data to date on the use of daratumumab prior to HSCT in multiple myeloma do not indicate reduced engraftment rates (66).

Another CD38-targeted naked antibody, isatuximab, is currently being investigated for safety and efficacy in a phase II study (ISAKIDS, ClinialTrials.gov: NCT03860844). This study is enrolling paediatric patients aged ≥28 days up to 18 years of age with B- and T-cell ALL.

## Conclusions

Children 4 years or younger affected with ALL are a fragile population both in terms of disease refractoriness, especially in the infant population, and a predisposition to relevant acute and long-term toxicities.

As relapse risk is still high, especially in the infants, better disease control is required. Possible interventions aiming at reducing the risk of relapse might include strategies to reduce MRD before HSCT, to improve the anti-leukaemic efficacy of the conditioning regimen, and to add therapeutic elements or immunomodulation in the post-HSCT phase.

Post-HSCT interventions such as earlier tapering of immunosuppression have been attempted. The use of small molecules, such as programmed death ligand 1 (PDL-1) inhibitors, has been attempted. The use of blinatumomab after transplantation in cases of MRD persistence or reappearance is currently under investigation within the FORUM trial. Novel targeted therapeutic options such as inotuzumab ozogamicin or CAR T cells might lead to a deeper level of remission upon HSCT.

Currently, treosulfan or busulfan used in combination with agents like thiotepa and fludarabine are probably the most frequently used conditioning regimens in this age group. Adding and/or substituting agents, such as etoposide or clofarabine, might lead to better outcomes. Although proven most efficacious in older children, as demonstrated by the FORUM trial, TBI is generally not used in those under 4 years because of the high risk of severe long-term side effects. However, at which age long-term side effects are comparable to older children is not known. The use of TBI for better leukaemia control is still controversial in the youngest patients. Doses lower than 12 Gy as well as innovative irradiation techniques with potentially reduced long-term toxicity might be worth exploring in controlled trials according to the same principle which drove novel investigations in older adults (67, 68).

The concept of fully replacing HSCT by long-lasting CAR T-cell therapy is appealing. It is well-known that at least a proportion of children, adolescents and young adults achieved long-term remission with this approach, but thoroughly designed prospective studies in larger international cohorts are needed to establish the proportion of patients who could possibly be spared HSCT.

Challenges are even greater in infants who are often very fragile and usually have a different ALL biology, often exhibiting *KMT2A* rearrangements. Worldwide collaborative groups studying this rare disease in children will provide the backbone of evidence required to drive improved outcomes, evaluating the various new developments described in this review.

## Author Contributions

All authors listed have made a substantial, direct, and intellectual contribution to the work and approved it for publication.

## Funding

The costs of this publication have been supported by the Children's Cancer Research Institute, Vienna, Austria.

## Conflict of Interest

JB has participated in advisory boards for Novartis, Janssen and Gilead, and speakers' bureaus for Novartis. AB has participated in advisory boards for Novartis and Amgen and speakers' bureau activities for Novartis, Amgen and Medac, and has received meeting support from Medac, Neovii and Novartis. The remaining authors declare that the research was conducted in the absence of any commercial or financial relationships that could be construed as a potential conflict of interest.

## Publisher's Note

All claims expressed in this article are solely those of the authors and do not necessarily represent those of their affiliated organizations, or those of the publisher, the editors and the reviewers. Any product that may be evaluated in this article, or claim that may be made by its manufacturer, is not guaranteed or endorsed by the publisher.
